# Biphasic pleural mesothelioma treated successfully with multimodal therapy: a case report

**DOI:** 10.1186/s44215-023-00077-8

**Published:** 2023-10-03

**Authors:** Kenshiro Omura, Ryuta Fukai, Tomoki Nishida, Nobuo Yamaguchi, Wataru Naitoh, Shinichi Teshima, Shunichi Tobe, Makoto Hibino, Fumihiro Tanaka, Masayuki Noguchi

**Affiliations:** 1 Division of Thoracic Surgery, Shonan Kamakura General Hospital, 1370-1, Okamoto, Kamakura, Kanagawa 247-8533 Japan; 2 Division of Pathology, Shonan Kamakura General Hospital, Kamakura, Kanagawa Japan; 3 Division of Respiratory Medicine, Shonan Fujisawa Tokushukai Hospital, Fujisawa, Kanagawa Japan; 4 Second Department of Surgery, School of Medicine, University of Occupational and Environmental Health, Kitakyushu, Fukuoka Japan; 5 Division of Pathology, NaritaTomisato Tokushukai Hospital, Narita, Chiba Japan

**Keywords:** Biphasic pleural mesothelioma, Macroscopic complete resection, Multimodal therapy

## Abstract

**Background:**

Pleural mesothelioma is an aggressive malignant tumor and has a poor prognosis. In particular, biphasic pleural mesothelioma is a less common histologic type, and successful outcomes are rare.

**Case presentation:**

A 60-year-old man was referred to our associated hospital because of dyspnea. Massive right pleural effusion and thickening of the entire right parietal pleura were revealed by radiological examination. After pleural biopsy, we diagnosed the patient’s tumor as biphasic pleural mesothelioma. The patient was admitted to our hospital for multimodal treatment. Two cycles of chemotherapy were initially administered with dramatic effects. Therefore, we decided to perform surgery and achieved a macroscopic complete resection. Postoperative chemotherapy was administered with no adverse events. No recurrence has been observed 11 months post-operation.

**Conclusions:**

We encountered a case of biphasic pleural mesothelioma that responded well to chemotherapy, enabling macroscopic complete resection.

## Background

Pleural mesothelioma (PM) is a highly aggressive tumor. The median survival of patients with the disease is about 1 year [1]. Biphasic pleural mesothelioma (BPM) is the least common histologic subtype, accounting for approximately 11% of PM cases [2]. Owing to its rarity, a concrete treatment strategy has not been established, especially for BPM. In addition, very few reports provide detailed descriptions of successful outcomes of patients with BPM. Here, we report a case of BPM that was successfully treated with multimodal therapy.

## Case presentation

**Fig. 1:**
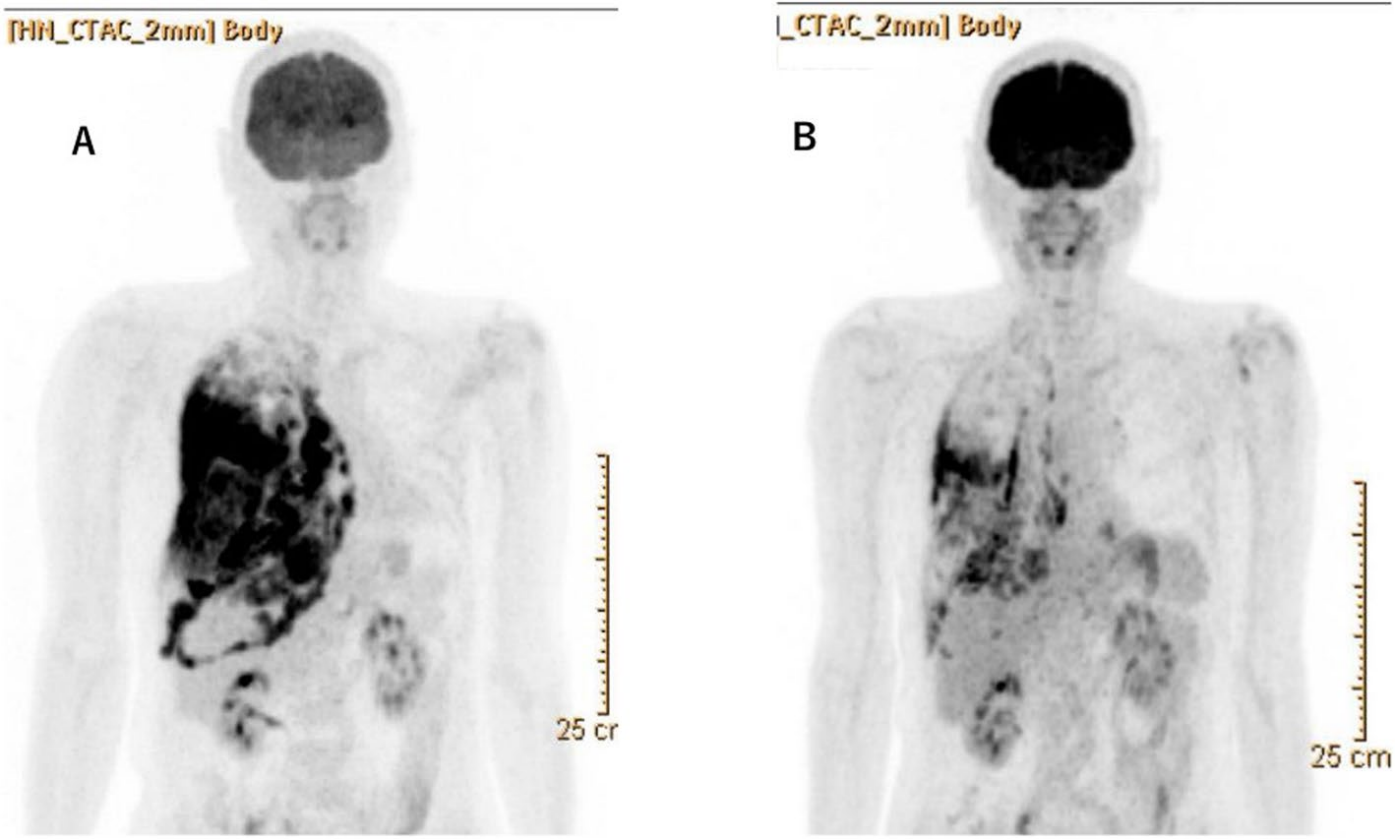
Findings of F-18 fluorodeoxyglucose (FDG) positron emission tomography (PET). **A** Before chemotherapy, FDG uptake was remarkable in the entire parietal pleura. **B** After 2 cycles of chemotherapy, FDG uptake was decreased and disappeared in some areas

**Fig. 2:**
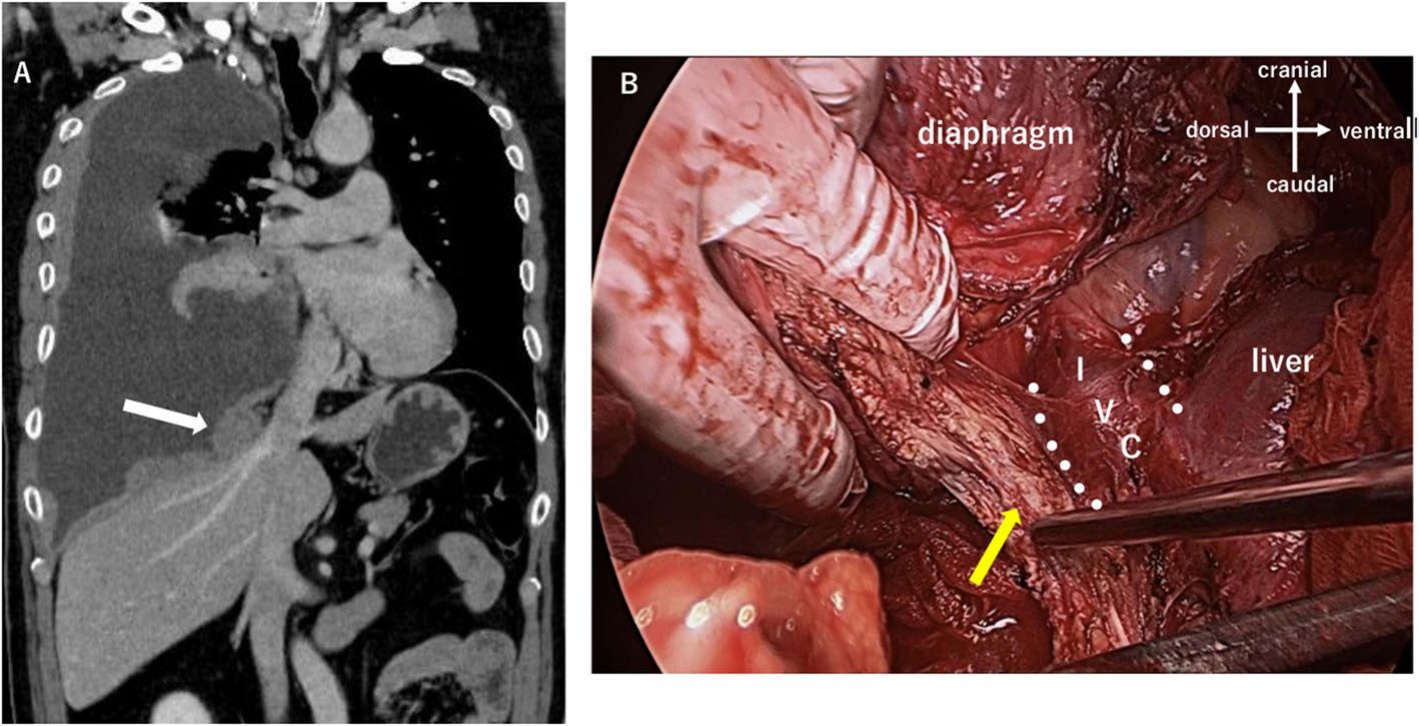
**A** Findings of contrast-enhanced computed-tomography (CT) scan. The tumor was very close to the inferior vena cava (IVC) and hepatic veins (white arrow). **B** Intraoperative photo viewed from the abdominal side. There was a large amount of tissue around the IVC, where the dissection was most difficult (yellow arrow)

A 60-year-old man with dyspnea was referred to our hospital by another. Chest X-ray showed massive right pleural effusion and computed tomography (CT) revealed thickening of the entire right parietal pleura. F-18 fluorodeoxyglucose (FDG) positron emission tomography (PET) revealed abnormal FDG uptake in the entire right parietal pleura (Fig. 1A). Since PM was highly suspected based on radiological findings, we performed a pleural biopsy by video-assisted thoracic surgery (VATS). Histologically, the tumor consisted of polygonal epithelial cells and spindle-shaped non-epithelioid cells. Therefore, we diagnosed the tumor as BPM. Except for the right pleura, no other sites, such as hilar lymph node and mediastinal lymph node, presented abnormal FDG uptake. In addition, the tumor was very close to the inferior vena cava (IVC) and hepatic veins, and there was severe pleural thickening at the same sites (Fig. 2A). Based on these findings, we considered that macroscopic complete resection (MCR) with certainty was difficult at first, but surgery would remain as a treatment option depending on the effect of chemotherapy. In our respiratory group discussion, at first, a doctor in the division of respiratory medicine considered using immunotherapy before surgery. However, we decided to use a standard regimen of chemotherapy as the first-line treatment and we planned to use immunotherapy in case of recurrence. After the discussion, we decided to perform surgery unless disease progression was noted after two cycles of chemotherapy and radiological findings indicated that MCR was impossible. If disease progression was noted after chemotherapy, surgery would not be indicated and we planned to continue only with chemotherapy. Then, patient was admitted to our hospital for multimodal treatment. The standard regimen of chemotherapy (cisplatin and pemetrexed) was administered for two cycles. After chemotherapy, we evaluated its effect along with the modified RECIST criteria and judged the partial response (PR) [3]. Surprisingly, FDG uptake in some parts of the right parietal pleura had completely disappeared (Fig. 1). Therefore, we proceeded to perform non-incisional pleurectomy/decortication (PD) [4], via a posterolateral thoracotomy at the sixth intercostal space. We resected the entire pericardium around the IVC, only leaving seam allowance and we made our best effort to expose as much of the IVC as possible on the caudal side (Fig. 2B). We also resected the entire diaphragm only leaving behind the peritoneum because tumor invasion to the diaphragm was apparent macroscopically. As a result, we were able to achieve MCR. Finally, the pericardium was reconstructed by attaching a 0.1-mm thick Gore-Tex sheet. The diaphragm was reconstructed by attaching a 2-mm thick Gore-Tex sheet two intercostal spaces higher than the normal position to reduce dead space. A 2-mm Gore-Tex sheet was sutured to the rib with non-resorbable polyfilament suture material. The postoperative course was generally uneventful, except for the need for pleural adhesion therapy using albumin and fibrin adhesive material due to a prolonged air leak. The patient was discharged 15 days after the surgery.

**Fig. 3:**
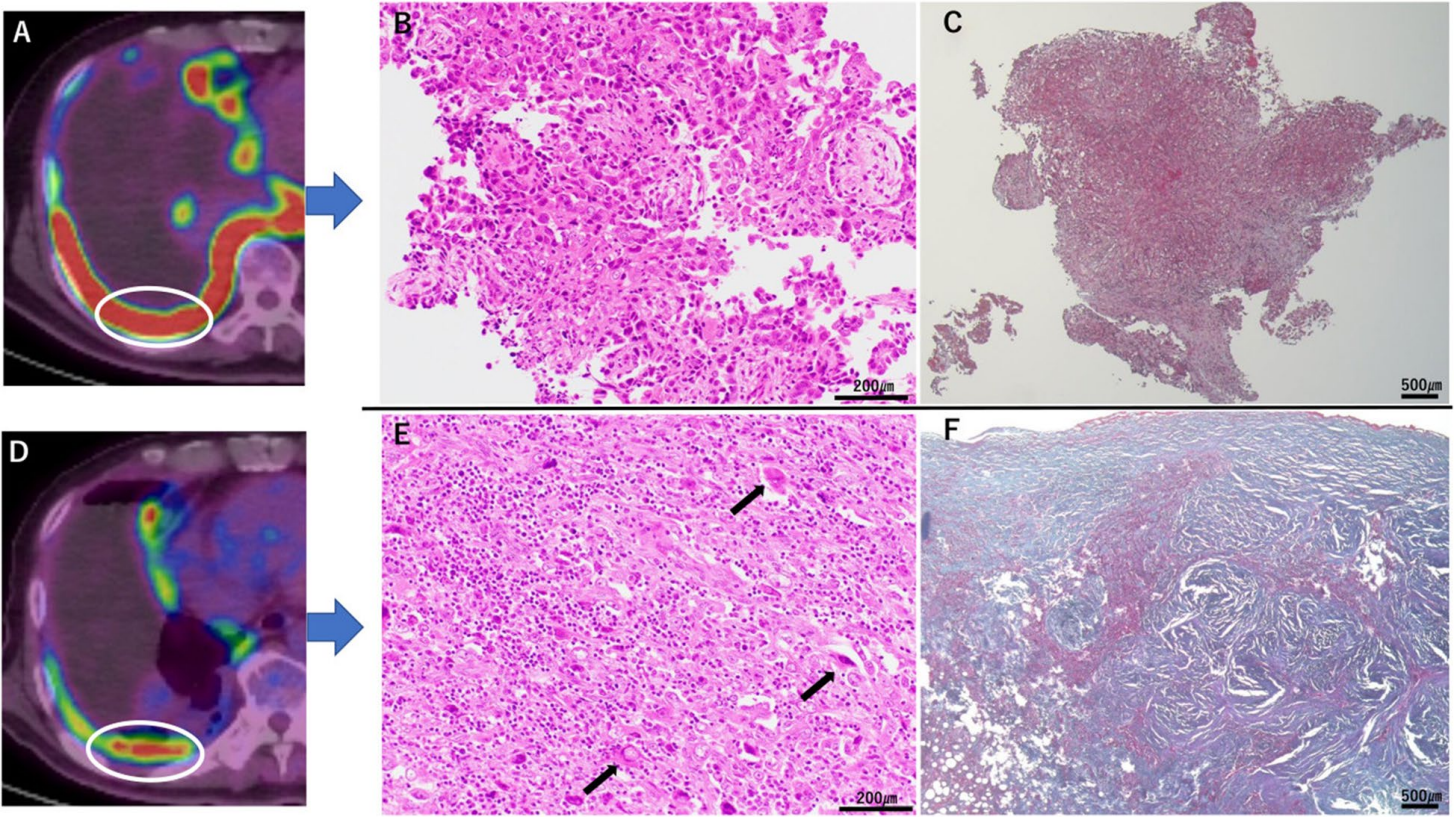
Histopathological findings. **A** Findings on positron emission tomography-computed tomography (PET-CT) around the biopsy lesion before chemotherapy (white circle). **B** Histologically the tumor was diagnosed as biphasic mesothelioma and many viable cells were observed (HE × 200). **C** There was no fibrotic area in the tumor (Elastica-Masson × 40).** D** Findings on PET-CT around the biopsy lesion after chemotherapy (white circle). **E** Surrounding the fibrotic area, tumor cells are scattered and most of them are degenerated and some cells had enlarged nucleus (black arrows) (HE × 200). **F** Blue colored fibrotic change was widely observed after chemotherapy (Elastica-Masson × 40)

The resected specimens were fixed in 10% buffered formalin and examined histologically. Macroscopically, the hard fibrotic area and soft area were mixed in the same tumor. Microscopically, we detected viable tumor cells in the diaphragm, but not in the pericardium and vessel sheath. To compare the effects of chemotherapy, we examined several sites differently based on the FDG-PET findings. The high-uptake area on FDG-PET (Fig. 3A) where the biopsy was performed, showed a highly cellular tumor and no fibrosis (Fig. 3B, C). However, after chemotherapy, the area showed reduced uptake of FDG-PET (Fig. 3D), and tumor cells infiltrated sparsely and appeared degenerated (Fig. 3E); extensive fibrosis was also observed (Fig. 3F). Most of the viable tumor cells were incohesive and their nuclei were enlarged and malformed (Fig. 3E). Therefore, we could not recognize their differentiation, whether it was epithelioid component or sarcomatoid component. The MIB-1 labeling index of the biopsy material (Fig. 3B) was 40% and that of the resected material (Fig. 3E) was 10%. To clarify characteristics of the infiltrating lymphocytes, we assessed the biopsy and surgical materials using CD3 and CD20 immunohistochemistry. As Fig. 3E indicated, the tumor tissue of the surgical material showed infiltration of small lymphocytes more than in the biopsy material (Fig. 3B). In both materials, lymphocytes positive for CD3 were more common than those positive for CD20.

The patient recovered three months after the surgery, and chemotherapy with the same regimen was restarted. Four cycles of postoperative chemotherapy were completed with no adverse events. Since then, there has been no recurrence of the disease for 11 months.

## Discussion and conclusions

In this report, we showed that we were able to select an appropriate treatment strategy and that MCR was possible after chemotherapy for BPM.

PM is a rare and aggressive malignant tumor with poor prognosis. Generally, it is difficult to obtain a complete cure for PM with only surgery or chemotherapy. Therefore, it is very important to consider a treatment strategy based on each patient’s condition. Many previous reports have suggested that multimodal therapy, including surgery, may be beneficial for PM patients [5]. Focusing on BPM patients, Meyerhoff et al. demonstrated that surgery slightly prolonged median survival [6]. However, the two major types of surgery, extrapleural pneumonectomy (EPP) and PD, are high-risk and highly-invasive procedures. Therefore, patient selection for surgery is particularly important.

Unfortunately, there are no concrete criteria for selecting patients to undergo surgery. In this case, the preoperative chemotherapy was effective. Some reports have demonstrated that postoperative survival was worse in patients who received neoadjuvant chemotherapy than in those who underwent immediate surgery [7]. In this case, because the tumor was very close to the IVC and the hepatic vein, we were not confident that we could perform MCR and wanted to avoid incomplete resection. We chose chemotherapy as the first line treatment which led to successful PD. Therefore, preoperative chemotherapy should be considered for marginally resectable tumors to increase the probability of achieving MCR.

We could not determine the reason for this positive response. Very few studies have investigated the factors associated with a good response to chemotherapy. Similarly, very few studies have compared the efficacy of chemotherapy based on histology. Histology is generally believed to be a prognostic factor, and patients with non-epithelioid tumor have worse survival [8]. However, this case suggests that chemotherapy may contribute to prognosis, even in BPM patients.

Many reports have identified the prognostic factors of PM. Recently, two reports have focused on BPM. Lococo et al. showed that independent variables, specifically performance status (0 or 1), percentage forced expiratory volume in 1 s (FEV1) > 80%, TNM, and a multimodal approach, affected long-term survival [9]. Furthermore, Lapidot et al. showed that age (70 years and younger), preoperative FEV1 > 80%, and adjuvant therapy were factors associated with overall patient survival through univariate analysis [10]. Based on these studies, our patient met all the above prognostic criteria; thus, we anticipated that the prognosis would be good. It is possible that these factors may overlap with the factors associated with a good response to chemotherapy, and if so, it may be necessary to consider such prognostic factors to determine treatment strategies.

In conclusion, we report a case of BPM that was successfully treated with multimodal therapy, including chemotherapy and surgery. Appropriate patient selection for surgery is believed to lead to a favorable prognosis.

## Data Availability

Not applicable.

## Abbreviations

PM: Pleural mesothelioma
BPM: Biphasic pleural mesothelioma
CT: Computed tomography
FDG: Fluorodeoxyglucose
PET: Positron emission tomography
VATS: Video-assisted thoracic surgery
IVC: Inferior vena cava
MCR: Macroscopic complete resection
PR: Partial response
P/D: Pleurectomy/decortication
EPP: Extrapleural pneumonectomy
